# Effects of Grinding Passes and Direction on Material Removal Behaviours in the Rail Grinding Process

**DOI:** 10.3390/ma11112293

**Published:** 2018-11-15

**Authors:** Shuyue Zhang, Kun Zhou, Haohao Ding, Jun Guo, Qiyue Liu, Wenjian Wang

**Affiliations:** Tribology Research Institute, State Key Laboratory of Traction Power, Southwest Jiaotong University, Chengdu 610031, China; lovingjanezsy@163.com (S.Z.); zhoukun@my.swjtu.edu.cn (K.Z.); dinghaohao520@163.com (H.D.); guojun@swjtu.cn (J.G.); liuqy@swjtu.cn (Q.L.)

**Keywords:** rail grinding, material removal behaviour, grinding passes, grinding direction

## Abstract

A three-dimensional finite element model of rail grinding was established to explore the effects of grinding passes and grinding direction on the material removal behaviour of grinding rails during the grinding process. The results indicate that as the number of grinding passes increases, a decrease in the grinding force reduces both the amount of removed rail material and the surface roughness. There is a decrease in the grinding ratio caused by the increase in the wear on the grinding wheel and the decreased removal of the rail material. When the grinding direction changes, the wear of the grinding wheel decreases, which is contrary to the increasing trend of the amount of removed rail material, the grinding ratio, the surface roughness and the grinding force.

## 1. Introduction

Rail grinding is a practical and economical maintenance technology to increase the lifetime of rails in existing railroad systems [1,2,3]. Rail grinding has widely been used in railways and has been involved in many studies in recent decades. Previous research in the field of rail grinding has mainly focused on the design of rail grinding patterns [1,4], the design of grinding rail profiles [5,6], temperature fields in the rail grinding process [7,8] and grinding train dynamics [9,10]. Many scholars have simulated and analyzed single abrasive particle grinding [11,12,13], which is an important means to understand the grinding process with the entire grinding wheel, as it can magnify the grinding degree and avoid the interference of other abrasive particles. However, it is nonetheless difficult to simulate rail grinding in the field with a complete 3D (three-dimensional) simulation model.

There are some studies that have focused on rail grinding experiments to investigate the removal behaviours. Gu et al. [14] analyzed the abrasive removal mechanism behaviours of rail materials under different rotational speeds of the grinding wheel. Uhlmann et al. [15] experimentally studied the processing results and the surface layer damage during the rail grinding process. He et al. [16] repeated rail grinding tests at the same angle on a grinding test bench to explore the change in material removal after each grinding pass. Furthermore, Zhi et al. [2] set up a predictive model of the rail grinding process using a distributed cutting grain approach to study the grinding pattern of multiple grinding wheels. It can be seen that the rail grinding in the field is a complicated tribological process, which includes cutting, material removal, surface deformation, wheel wear, and other factors. Many factors significantly affect the material removal behaviours on the interface between the rail and the grinding wheel, which affects the grinding performance.

**Figure 1:**
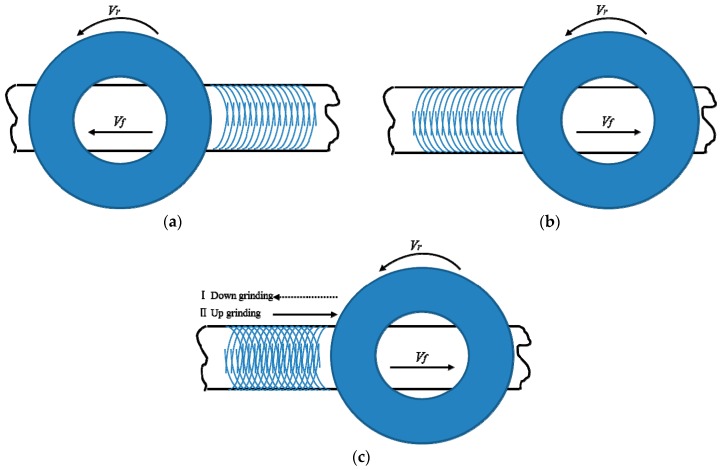
Schematic diagram of the grinding band after rail grinding: (**a**) down grinding; (**b**) up grinding; and (**c**) up grinding after down grinding.

Many researchers have studied the material removal behaviours during the grinding process. Yosiffon et al. [17] used grinding wheels with different hardnesses and abrasive particle sizes to grind stainless steel and found the changes in the abrasive wear with metal removal rate and noted that the main form of abrasive wear is abrasive peeling. Agarwal et al. [18] revealed that the material removal mechanism in the SiC grinding process is due to crystal displacements caused by grain boundary micro-cracks. Cheng et al. [19] studied the significant influence on the surface roughness caused by vibrations in the grinding process. Maksoud et al. [20] assumed that the parameters obey an exponential distribution law and used grey theory to predict the grinding force. It should be pointed out that the number of grinding passes and grinding direction both significantly influence the material removal behaviours at the interface between the rail and the grinding wheel. In Figure 1, a grinding band occurred after rail grinding on the rail surface, and the surface morphology is superimposed by the individual motion trajectory of the grinding wheel particles involved in the process, which can be simplified as a set of curves. Changes in the orientation of these curves are caused by changes in the grinding direction, which is realized by the running direction of the grinding train and is regarded as down-grinding and up-grinding, as shown in Figure 1a,b, respectively. The reciprocating grinding process is shown in Figure 1c, and the total number of grinding passes is three in this simulation study, as is common in the field. Since some material removal behaviours, such as the wear of the grinding wheel, the grinding ratio and the grinding force, are difficult to analyze experimentally, it is necessary to study the material removal behaviours during rail grinding in the field using simulations. Meanwhile, the effects of the number of grinding passes and the grinding direction on the material removal behaviours at the interface between the rail and the grinding wheel have seldom been analyzed.

In this study, a three-dimensional model of rail grinding was established to perform finite element (FE) simulations of the field reciprocating rail grinding process. The grinding material removal behaviours, including the wear of the grinding wheel, the removal of rail material, the grinding ratio, the surface roughness and the grinding force, in every grinding pass and different grinding directions, are analyzed in detail.

## 2. Simulation Details

### 2.1. Simulaiton Model

**Figure 2:**
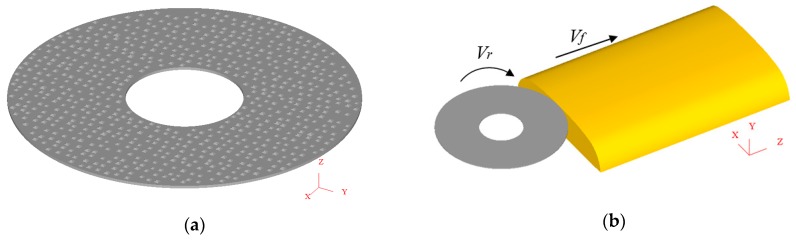
3D model of rail grinding in the field showing (**a**) the grinding wheel and (**b**) rail grinding.

According to reference [21], Han has previously established a 3D model of a 16# grinding wheel, the process of which is described below. First, a portable digital microscope (DM, KEYENCE, Osaka, Japan) is used to measure the end surface morphology of the grinding wheel, and it is deduced that the surface density of the abrasive grain is 0.27 mm^−2^. Then, after the analysis of the data from the JB-6C surface roughness measuring instrument (Wilson, Guangzhou, China), it was found that the distribution law of the outburst height of the abrasive grain obeys a normal distribution. The size of the abrasive particle is approximately 1000 microns based on the virtual lattice method. Next, the *X*, *Y* and *Z* coordinates of each abrasive particle that were calculated using compiled MATLAB functions are imported into the AutoCAD software (v2014, Autodesk, San Rafael, CA, USA) as the 3D center point of each abrasive particle. The abrasive particles were then placed at the corresponding points in the simulation domain, and the shape of each abrasive particle is simplified as a pyramid. Finally, after simplifying the grinding wheel bond as a cylinder and assembling it with the abrasive particles, a grinding wheel model is established. Following consideration of the convenience of pre-processing settings and the improvement of the solving speed, this paper reduced the thickness of Han’s grinding wheel bond model from 10 mm to 0.5 mm without affecting the simulation results (Figure 2a). Then, the part 2 mm from the top of the rail head is used to establish the rail model, and the 3D model of rail grinding comprises the combination of the rail and the grinding wheel model, as shown in Figure 2b.

### 2.2. Simulation and Material Parameters

Detailed parameters must be confirmed to simulate the reciprocating rail grinding process. The normal grinding pressure is one of the key parameters in rail grinding, but the interaction between the grinding wheel and the rail is set by the grinding depth in the simulation study. So, to obtain the value of the grinding depth, the width of the grinding band is computed from six groups of repeated reciprocating rail grinding experiments from the rail grinding simulation testing apparatus, which can simulate rail grinding in the field. The experimental parameters are given in Table 1, and the simulation parameters are the same as those used in the field. After the experiment, the grinding depth is calculated by the width of the grinding band (Table 2), and the deviations of the six groups of experiments are listed in Table 2. The formula for the grinding depth is as follows.

(1) $$D=R-\sqrt{R^{2}-{\left(\frac{L}{2}\right)}^{2}}$$

where *L* is the width of the grinding band in Table 2, *D* is the grinding depth, and *R* is the radius of the rail head at 300 mm.

Table 2 shows that the grinding depth decreases with the increase in the number of grinding passes. This is because the grinding wheel is subjected to the same normal force of 1800 N at each grinding pass, but the area of the rail head involved in the grinding process may increase with the number of grinding passes due to the rail head consisting of five arc areas. Therefore, the normal intensity of the pressure on the rail decreases, the normal deformation of the rail decreases, and the depth of grinding decreases.

In the rail grinding process, the main component of the grinding wheel is Al_2_O_3_, and the rail material is U71Mn (Chinese brand, Pangang Group Company Limited, Panzhihua, China) steel. The physical properties of these materials are given in Table 3 and Table 4, respectively. In addition, since the hardness of the grinding wheel is much greater than that of the rail material, the grinding wheel is set as a rigid body while the rail material is set as a plastic body. The constitutive relation of the materials refers to the relationship between the flow stress and the thermodynamic parameters, such as the temperature, strain and strain rate, which are used to characterize the dynamic responses of the materials during deformation. Under the action of external forces during rail grinding, the rail material will undergo elastic deformation and plastic deformation until fracturing at high temperature, large strain and a large strain rate [22,23,24]. To better simulate the rail grinding process, Johnson–Cook constitutive models are widely used because they are relatively simple (with five parameters) and numerically robust. The constitutive equation of the Johnson-Cook material is as follows:

(2) $$\sigma =(A+B\varepsilon^{n})[1+C\ln(1+\frac{\dot{\varepsilon }}{{\dot{\varepsilon }}_{0}})][1-{\left(\frac{T-T_{r}}{T_{m}-T_{r}}\right)}^{m}]$$

where *A*, *B*, *n*, *C* and *m* represent the yield strength, hardening modulus, hardening coefficient, strain sensitivity coefficient and thermal softening coefficient under a static state, respectively. ε is the equivalent plastic strain, $\dot{\varepsilon }$ is the equivalent plastic strain rate, ${\dot{\varepsilon }}_{0}$ is the reference strain rate, *T_r_* and *T_m_* are the room temperature and material melting point temperature, respectively, and *T* is the temperature of the workpiece [25]. The constitutive parameters assigned to rail materials are given in Table 5.

**Figure 3:**
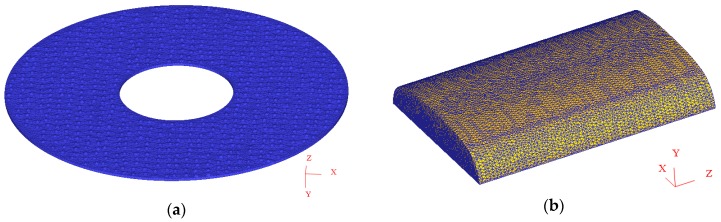
Meshed model of rail grinding in the field showing (**a**) the grinding wheel and (**b**) the rail.

The finite element meshed model of the grinding wheel and rail was established using the finite element software DEFORM-3D (v10.2, Science Forming Technology Corporation, Clumbus, OH, USA) using a self-contained hybrid tetrahedron element. This type of element is commonly used since fewer element nodes can better describe the geometry model and can automatically divide the element with a better shape quality by the linear characteristic. The grinding wheel and rail meshed model are shown in Figure 3a,b respectively. The grain mesh consists of 200,000 elements with minimum element size of 0.5 mm while the rail mesh model is discretized with 250,000 elements, giving a minimum element size of about 0.3 mm.

To simulate rail grinding in the field as precisely as possible, the fixed rail is ground by the rotary grinding wheel, which moves along the length direction of the rail. Therefore, in the pre-processing of the DEFORM-3D software, the angular velocity rotating around the *Y* axis, which is the geometric center of the grinding wheel, is set as the grinding wheel rotational speed, and the translational velocity along the *Z* axis is set as the feed speed of the grinding wheel. Meanwhile, the translational motion along the *X* and *Y* axes and the rotational motion along the *X* and *Z* axes are all restrained. Furthermore, the full freedom constraint is applied to the bottom of the rail. In addition, the relative position of the grinding wheel and the rail is placed according to the grinding depth, and the contact between the grinding wheel and the rail is assigned as a sliding contact with a constant friction coefficient of 0.52. Therefore, in this study, three main process parameters are considered: the rotational speed (*n*), feed speed (*v_f_*), and grinding depth (*ap*). An illustration of these process parameters is presented in Figure 2b. According to the experiment, the values of the rotational speed and the feed speed are 3600 r/min and 83.33 m/min, respectively, and the grinding depth of each grinding pass is given in Table 2.

A tool wear model mainly describes the relationship between the tool volume loss rate and the cutting surface temperature, the relative sliding speed, the contact pressure and the cutting condition parameters. The two common wear models are the Archard model [26] and the Usui model [27]. The Archard model is mainly used to analyze the wear condition of soft materials in the friction process between hard and soft materials, and the Usui model is mainly used in continuous machining processes. Thus, the wear model based on sliding wear proposed by Usui has been utilized in this study, and the *a* and *b* parameters in the Usui wear model are set as 10^−9^ and 10^3^, respectively, according to the experimental values, and the hardness value is assigned to the grinding wheel. 

**Figure 4:**
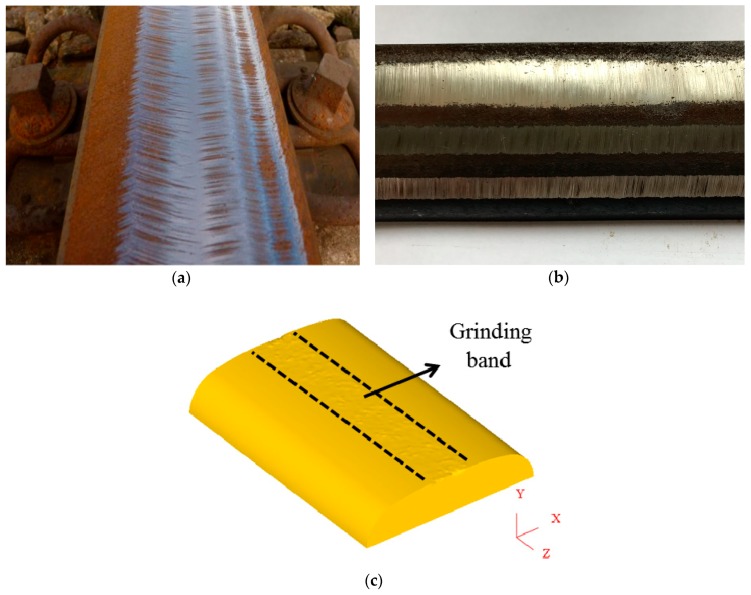
Verification of the grinding band between (**a**) the field, (**b**) the experiment and (**c**) the simulation.

The wear scar morphology and the width of the grinding band on the rail obtained from the rail grinding experiment on the rail grinding simulation testing apparatus (Figure 4b) is in good agreement with those in the field (Figure 4a), which shows that the rail grinding experiment can well simulate the grinding effect of the rail and grinding wheel [28]. Therefore, the data and analysis results from the experiment can well characterize rail grinding in the field.

**Figure 5:**
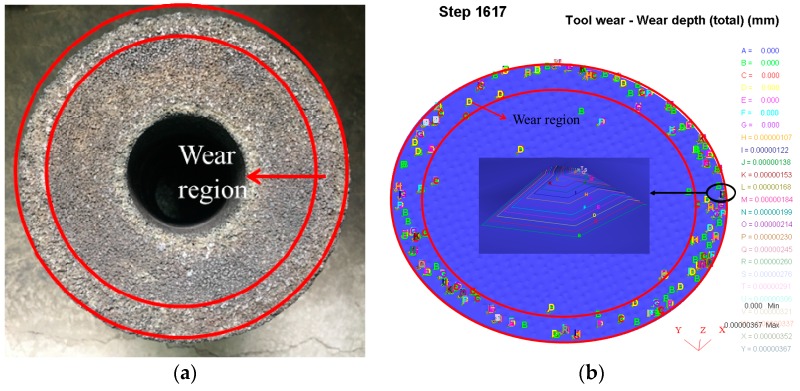
Verification of the wear region of the worn grinding wheel between (**a**) the experiment and (**b**) the simulation.

Figure 4 shows the grinding band is in the middle of the rail after rail grinding in the simulation, which is similar to that in the field and the experiment. In Figure 5, the worn grinding wheel in the experiment and simulation are compared, and it is found that the wear region of the wheel is quite similar, which is mainly observed on the grinding wheel edge. These two figures indicate that the simulation in this study is real and effective.

## 3. Results

The grinding pass changes three times while the common grinding directions of the three passes in the field are down-grinding, up-grinding and down-grinding, respectively. To exclude the particularity of a single simulation process, two groups of comparative simulation processes are carried out to study the grinding material removal behaviours after changing the grinding direction. Based on the commonly used grinding direction in three continuous grinding passes, in group A, the grinding direction of the second grinding pass is changed from up-grinding to down-grinding, and for group B, the grinding direction is changed from down-grinding to up-grinding in the third grinding pass. Both the grinding directions of two continuous grinding passes in the two groups change from being different to being the same.

### 3.1. Wear Amount for the Grinding Wheel

In the grinding process, the grind capacity of the grinding wheel significantly influences its working state. As the grinding time is extended, the cutting ability of the grinding wheel decreases, and various grinding defects continually appear, which means the grinding process cannot continue. At this point, the grinding wheel must be repaired to restore the normal grinding state. The actual grinding time between two dressings is referred to as the lifetime of the grinding wheel, which is an important factor influencing the grinding effect. However, it is very difficult to experimentally investigate the effect of grinding wheel wear. Therefore, in this study, the effect of tool wear is investigated using the finite element software DEFORM-3D.

The grinding wheel after rail grinding simulation is shown in Figure 5b obtained from the post-processing of the DEFORM-3D software. Letters from “*A*” to “*Z*” in Figure 5b represent the contour level of the wear depth of the entire grinding wheel at each step. Each letter corresponds to a numeric value of wear depth. The grinding wheel wear is usually characterized by the volume of the worn grinding wheel material. Therefore, in this paper, the wear amount volume *V* of a single abrasive grain is approximately equal to the product of the wear superficial area *S* and the wear depth *h* of each abrasive grain. The wear superficial area *S* of one abrasive grain is approximately equal to its superficial area (unified as *S* = 1 mm^2^ after a simplified calculation of a pyramid), and the wear depth *h* of one abrasive grain approaches the maximum contour level of wear depth. Then, the numerical value of the wear volume of a single abrasive grain is approximately equal to its maximum contour level of wear depth. Therefore, the numerical value of the wear volume of the entire grinding wheel is roughly the sum of the maximum wear depth of all abrasive grains.

**Figure 6:**
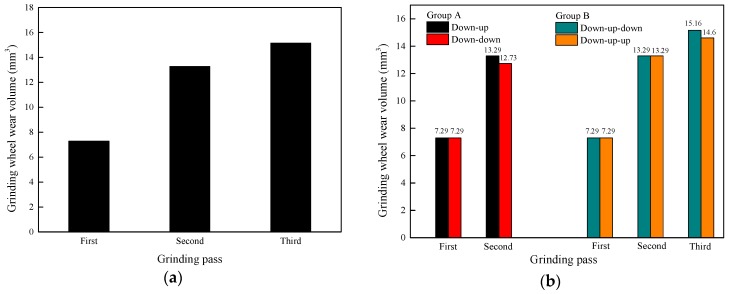
Change in the grinding wheel wear with (**a**) the number of grinding passes and (**b**) a change in the grinding direction.

The increment of the grinding wheel wear volume in each grinding pass is illustrated in Figure 6a. The wear volume increases with the increase in the number of grinding passes. This phenomenon appears on account of the continuous increasing wear of the grinding wheel during the grinding process. Since the wear is a cumulative process, the increment of the wear of the grinding wheel increases, although the grinding distance is the same for each grinding pass. The grinding wheel needs to be replaced after grinding for a certain time. In Figure 6b, the wear of the grinding wheel is reduced little after changing the grinding direction. As the grinding directions of two continuous grinding passes are the same, the rail surface morphology in the grinding band after grinding is less interlaced because the sets of curves on the rail surface between the two continuous passes are in the same orientation, so wear action of the grinding wheel affected by the rail is weakened and the volume of grinding wheel wear decreases.

### 3.2. Volume of Removed Rail Material

**Figure 7:**
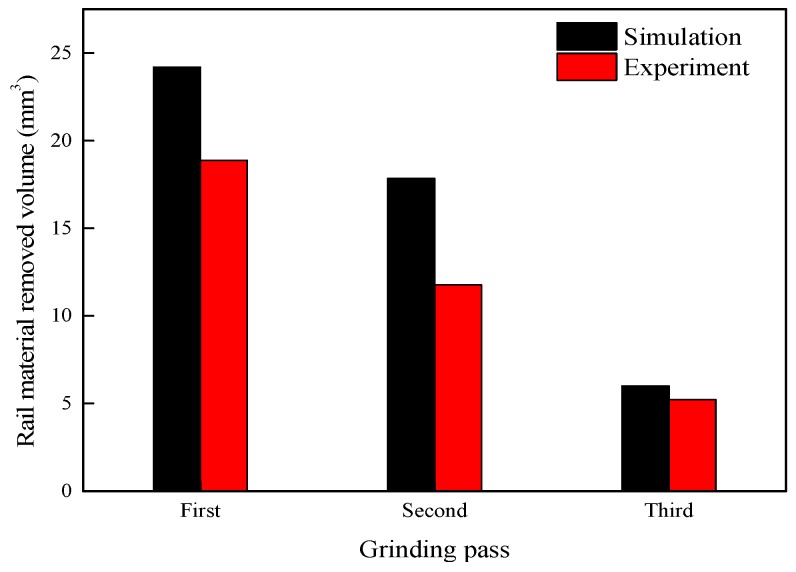
Change in the volume of removed rail material with the number of grinding passes in the simulation and the experiment.

The volume of removed rail material is caused by the removal action of the surface material affected by the grinding wheel. Variations in the increment of the volume of removed rail material with the grinding distance are calculated from the variation of the rail volume versus time, which is obtained from the post-processing of DEFORM-3D. As shown in Figure 7, with the increase in the number of grinding passes, the increment of the volume of removed rail material has a decreasing trend. Moreover, in Figure 7, the changing trend of the removed volume of rail material from the experiment is the same as the simulation result, and the numerical values of the simulation and experiment are on the same scale.

**Figure 8:**
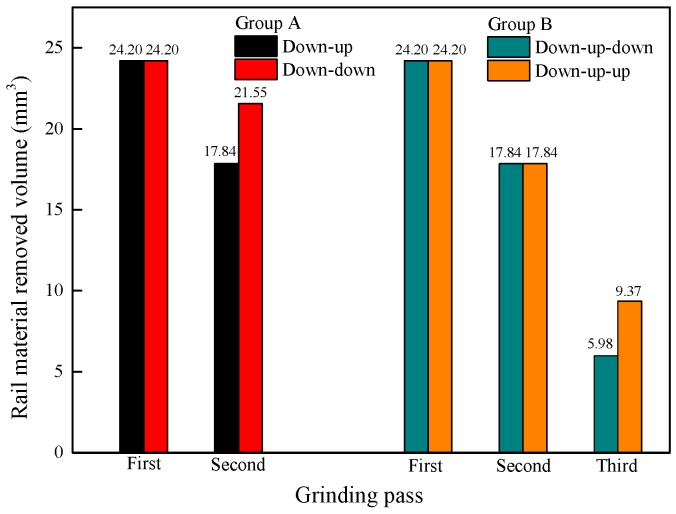
Change in the volume of removed rail material with a change in the grinding direction.

Figure 8 shows that as the grinding direction changes, the volume of removed rail material increases. This is because the rail surface topography changes to the same orientation after changing the grinding direction while the changed trend of the rail surface topography is in a different orientation as before. Thus, it is easier to remove rail material after changing the grinding direction than before. Therefore, the volume of removed rail material increases after changing the grinding direction.

### 3.3. Grinding Ratio

**Figure 9:**
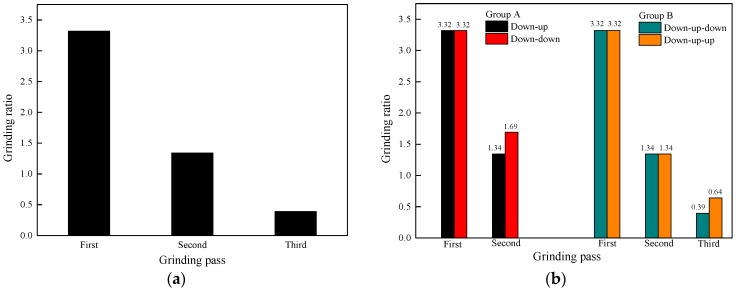
Change in the grinding ratio with (**a**) the number of grinding passes and (**b**) a change in the grinding direction.

The grinding ratio is the proportion of the volume of removed workpiece material to the volume of abrasive wear, which reflects the cutting efficiency of the grinding wheel. The grinding ratio decreases with the increase in the number of grinding passes, as shown in Figure 9a. This is attributed to the weakening of the grinding performance of the grinding wheel and the shortening of its service life. The variation trend of the grinding ratio is due to the performance of the grinding wheel gradually weakening with the grinding process; thus, the volume of removed rail material is reduced under the same volume of wear from the grinding wheel.

Grinding ratio increases with the change in the grinding direction, as shown in Figure 9b. This variation trend of the grinding ratio after the change in the grinding direction implies an enhanced performance of the grinding wheel. This is because the rail material is more easily removed and the wear effect of the grinding wheel is reduced after the grinding direction is changed in two continuous grinding passes.

### 3.4. Surface Roughness of the Grinding Rail

**Figure 10:**
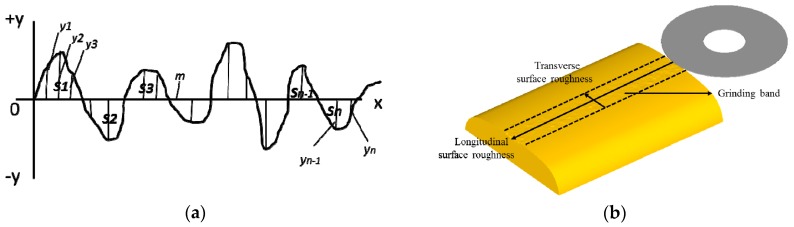
Schematic diagram of the surface roughness: (**a**) definition and (**b**) cross section direction.

Regardless of the processing method used, tiny bumps and uneven marks on the surface of the processed parts are inevitable, and alternating peaks and valleys will also appear. This phenomenon defines the surface roughness of the processed parts. There are three computing methods for the surface roughness calculation, namely, *Ra* (arithmetical mean roughness), *Ry* (maximum peak roughness) and *Rz* (ten-point mean roughness). Of these, *Ra* is the most commonly utilized. In Figure 10a, *m* is the midline of the surface profile of the parts, and the area between the midline *m* and the two sides of the contour line is equal. The average arithmetic deviation of the absolute value of the distance of each point on the contour to the midline m within a certain measuring length range is *Ra*, as shown in Equation (3).

(3) $$S_{1}+S_{3}+\ldots +S_{n-1}=S_{2}+S_{4}+\ldots +S_{n}$$

The experimental surface roughness is measured directly from the JB-6C surface roughness measuring instrument, but the theoretical calculation of surface roughness is seldom found in simulation studies. In the simulation performed for this paper, the surface roughness of the rail is computed after the grinding process, which is shown in Figure 10b. The surface roughnesses in both the transverse and longitudinal directions are studied and analyzed. Five cross-sections are selected at the same distance, both in the transverse and longitudinal directions, and each cross-section is inverted into AutoCAD software. Then, the multi-section tool is used to draw the cross-section contour, and the midline *m* is found and the surface roughness is calculated according to the *Ra* method mentioned in Equation (3). The average values of the surface roughness of the five sections in the two directions are considered as the surface roughness values in these two directions. 

**Figure 11:**
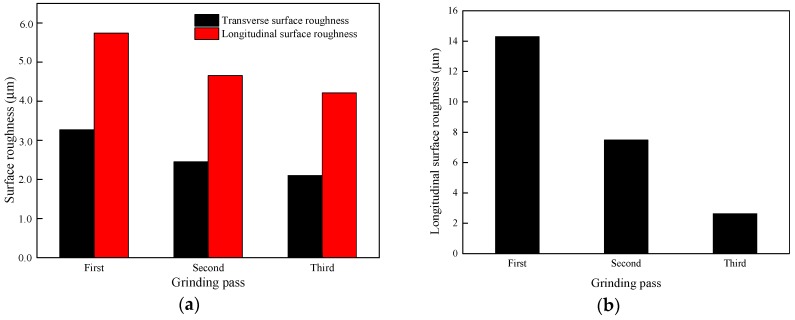
Change in the surface roughness with the number of grinding passes in (**a**) the simulation and (**b**) the experiment.

The results of the surface roughness in two directions in Figure 11a indicate that with the increase in the number of grinding passes, the surface roughness decreases. During the second grinding pass, the alternating peaks and valleys on the rail surface formed after the first grinding pass become smooth under the removal by the grinding wheel, so the surface roughness decreases. Figure 11a also shows that the longitudinal surface roughness is always higher than in the transverse direction. Meanwhile, the comparison between the simulation and the experiment of the surface roughness in Figure 11a,b shows that the changing trend is the same and the numerical values are on the same scale.

**Figure 12:**
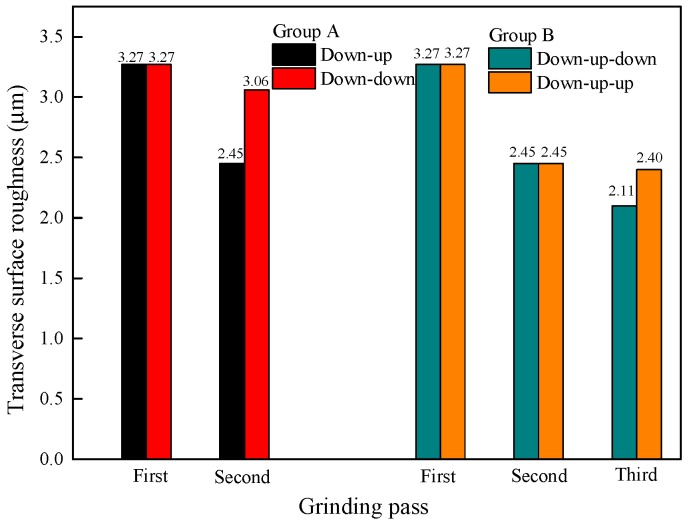
Change in the surface roughness with a change in the grinding direction.

The results in Figure 12 indicate that after changing the grinding direction, the surface roughness increases, which means that the quality of the rail surface after grinding becomes worse. Since the grinding direction of the second grinding pass differs from that of the first pass, it is easier to remove or even grind smooth the remaining burrs on the rail surface after the first grinding pass. However, the convex part of the first grinding pass is extruded in the same direction when the second pass is in the same grinding direction as the first pass, leading to worse surface quality and an increase in the surface roughness from the obvious deformation of the bulge after changing the grinding direction.

### 3.5. Grinding Force

The grinding force is the sum of the cutting force and the friction force during grinding. This force has an effect on the formulation of the grinding process and the damage of both the grinding surface and the subsurface. Additionally, an important variable is the evaluation of the grinding ability of the grinding wheel. Since the magnitude and the direction of the grinding forces are constantly changing during grinding, the grinding forces are generally classified into three perpendicular forces: the normal force (*F_n_*), tangential force (*F_t_*) and axial force (*F_a_*). In this study, the variation of the overall loads of the grinding wheel in the three directions versus time is obtained from the post-processing using DEFORM-3D, which is regarded as the variation of the grinding force with the rail grinding process. 

**Figure 13:**
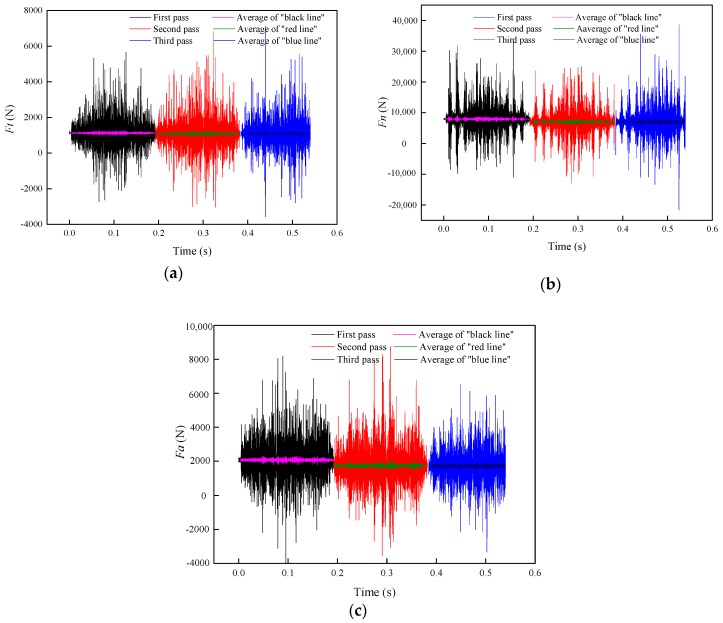
Change in the grinding force with the number of grinding passes: (**a**) *F_t_*; (**b**) *F_n_*; and (**c**) *F_a_*.

As shown in Figure 13, the grinding forces in the three directions remain nearly unchanged in general, and the grinding force of the second pass is slightly lower than that of the first pass. This is because the grinding force is positively correlated with the grinding depth. With the increase in the grinding depth, the contact arc length of the grinding wheel and rail, the grinding thickness of a single abrasive particle, and the number of abrasive particles involved in the grinding process all increase, which increases the grinding force. Table 2 indicates that the depth of the second grinding pass is slightly reduced, while the change in the grinding depth of the third pass is almost negligible. Therefore, the grinding force of the second pass is slightly reduced while the grinding force of the third pass is almost the same as that of the second pass.

**Figure 14:**
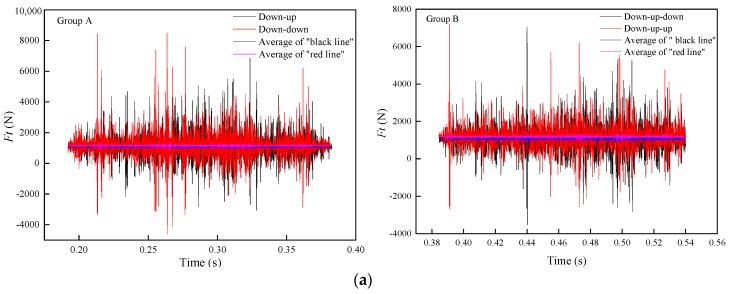

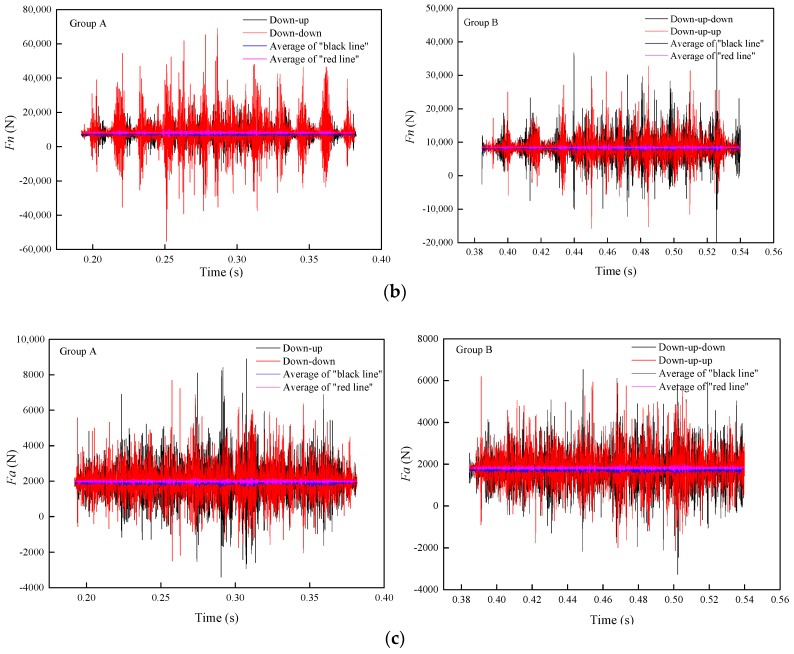
Change in the grinding force with a change in the grinding direction: (**a**) *F_t_*, (**b**) *F_n_*, and (**c**) *F_a_*.

Figure 14 shows the changes in *F_t_*, *F_n_*, and *F_a_* before and after changing the grinding direction. The results suggest that the grinding force is slightly increased after changing the grinding direction. Even the grinding depth after changing the grinding direction in the same grinding pass is almost the same, but changing the orientation of the material on the rail surface also has a certain effect on the grinding force. The direction of the grinding force is the same as the change trend of the material on the rail surface. Therefore, the grinding force increases while the direction of the grinding force in the second grinding pass is the same as that in the first grinding pass.

## 4. Discussion

This work presents a 3D finite element-based simulation of field reciprocating rail grinding of U71Mn rails with an Al_2_O_3_ grinding wheel. The reciprocating grinding of rails is achieved by changing the grinding direction between down-grinding and up-grinding. In addition, the material removal behaviours on the interface between the rail and the grinding wheel play a vital role in the analysis of the rail grinding process. Since the simulation results of this paper are similar to those from the experiment, and the experimental data are in good agreement with the field data, it is concluded that the rail grinding simulation in this study can effectively model the rail grinding in the field, and the method of analyzing the material removal behaviours on the interface between the rail and grinding wheel is feasible. It is worth noting that, because the numerical value of grinding wheel wear volume is estimated in the simulation analysis, a modified coefficient is needed according to further analysis of wheel wear in a reciprocating rail grinding experiment. A modified coefficient for the grinding ratio can then also be presented.

Due to the relatively large volume of removed rail material required from rail grinding in the field, multiple grinding passes are needed [4]. The results of this study show that with the increase in the number of grinding passes, the wear of the grinding wheel increases, while the volume of removed rail material, the grinding ratio, the surface roughness and the grinding force all decrease. It is obvious that the number of grinding passes significantly influences the material removal behaviours on the interface between the rail and the grinding wheel. Hence, it is necessary to consider the effect on the material removal behaviours of the increase in the number of grinding passes in rail grinding. It is also prudent to consider whether to increase the number of the grinding passes blindly to increase the volume of removed rail material.

Furthermore, to break through the existing grinding direction of reciprocating rail grinding, this paper studies the changes in the material removal behaviours at the interface between the rail and the grinding wheel after changing the grinding direction. Then, it is determined whether the current grinding direction is the optimal choice. The results of this study indicate that after changing the grinding direction, the wear of the grinding wheel decreases, while the volume of removed rail material, the grinding ratio, the surface roughness and the grinding force all increase. Since the purpose of rail grinding is to repair irregularity on the rail surface and obtain better surface quality, the resulting surface roughness must be considered. As the surface roughness increases after changing the grinding direction, there is no requirement to change the grinding direction in rail grinding in the field. When the analysis focuses on prolonging the service life of the grinding wheel, the wear on the grinding wheel, which is reduced after changing the grinding direction, is an important parameter. Therefore, changing the grinding direction is a means to prolong the service life of the grinding wheel. Meanwhile, these results can also be applied to the selection of the grinding direction for any grinding process other than rail grinding, in addition to some cutting processes. 

The study results suggest that the changing trend of the volume of removed rail material, the grinding ratio, the surface roughness and the grinding force are the same, while the opposite trend is seen for the grinding wheel wear. This is because the rail material is easier to remove when the grinding force is increased, so the volume of removed rail material increases with the increase in the grinding force. Then, the surface morphology of the rail becomes more complicated after an increase in the volume of removed rail material, which leads to an increase in the surface roughness. Meanwhile, as the tendency of the volume of removed rail material and the wear of the grinding wheel are opposite, the grinding ratio is positively correlated with the volume of removed rail material.

## 5. Conclusions

(a) A finite element model of rail grinding in the field was established with DEFORM-3D software. The simulation results are used to evaluate the grinding material removal behaviours in the rail grinding process.

(b) The grinding passes significantly affect the material behaviours at the interface between the rail and the grinding wheel. With the increase in the number of grinding passes, the grinding force is decreased and reduces both the volume of removed rail material and the surface roughness. The increase in the wear on the grinding wheel and a decrease in the removed rail material cause a fall in the grinding ratio.

(c) After changing the grinding direction, the grinding force increases and intensifies both the volume of removed rail material and the surface roughness. The decrease in the grinding wheel wear and the increase in the volume of removed rail material cause a rise in the grinding ratio.

(d) There is a positive correlation among the volume of removed rail material, the grinding ratio, the surface roughness and the grinding force, which are negatively correlated with the wear of the grinding wheel.

Further work on the material behaviours for the rail grinding process should be performed with a comparison between the actual and simulation results, a study on grinding wheel wear volume in a rail grinding experiment, a study on other types of inter-surface material removal behaviour, such as surface residual stress, and a study on the material removal behaviour in the temperature field.

## Figures and Tables

**Table 1:** The experimental parameters.

Normal Force (N)	Rotational Speed (r/min)	Feed Speed (mm/min)	Grinding Angle (°)	Grinding Mode
1800	3600	83.33	2	down-up-down

**Table 2:** Width of the grinding band and grinding depth.

Grinding Pass		Ⅰ	Ⅱ	Ⅲ
Width (mm)	Average	12	13.66	14.3
	Deviation	0.1	0.3	0.35
Depth (mm)	Average	0.06	0.01776	0.007456
	Deviation	0.05	0.15	0.175

**Table 3:** Main physical property parameters of the rail material.

Rail Material	Young’s Modulus (MPa)	Poisson’s Ratio	Thermal Conductivity (N·s^−1^·°C^−1^)	Heat Capacity (N·mm^−2^·°C^−1^)
U71Mn	2.06754 × 10^5^	0.3	Change with temperature	

**Table 4:** Main physical property parameters of the grinding wheel material.

Grinding Wheel Material	Young’s Modulus (MPa)	Poisson’s Ratio	Density (kg·m^−3^)
Al_2_O_3_	3.7 × 10^5^	0.22	3960

**Table 5:** Johnson-Cook parameters of the rail material.

*A* (MPa)	*B* (MPa)	*n*	*C*	*m*
792	510	0.26	0.014	1.03
